# Positive Association of Metabolic Syndrome with a Single Nucleotide Polymorphism of Syndecan-3 (rs2282440) in the Taiwanese Population

**DOI:** 10.1155/2018/9282598

**Published:** 2018-01-30

**Authors:** Betty Chia-Chen Chang, Lee-Ching Hwang, Wei-Hsin Huang

**Affiliations:** ^1^Department of Family Medicine, Mackay Memorial Hospital, Taipei City, Taiwan; ^2^Mackay Medical College, New Taipei City, Taiwan

## Abstract

**Background/Purpose:**

Metabolic syndrome (MetS) poses a major public health burden on the general population worldwide. Syndecan-3 (SDC3), a heparin sulfate proteoglycan, had been found by previous studies to be linked with energy balance and obesity, but its association with MetS is not known. The objective of this study is to investigate whether SDC3 polymorphism (rs2282440) is associated with MetS in the Taiwanese population.

**Methods:**

Genotypes of SDC3 polymorphism (rs2282440) were analyzed in 545 Taiwanese adult subjects, of which 154 subjects had MetS.

**Results:**

Subjects with SDC3 rs2282440 TT homozygote had higher frequency of MetS than those with CC or CT genotype (*p* = 0.0217). SDC3 rs2282440 TT homozygote had a 1.96-fold risk of being obese and 1.8-fold risk of having MetS (with CC genotype as reference). As for the individual components of MetS, subjects with SDC3 rs2282440 TT homozygote were more likely to have large waist circumference and low high-density lipoprotein cholesterol (OR = 1.75 and OR = 1.84, resp.).

**Conclusion:**

SDC3 rs2282440 polymorphism is positively associated with MetS in the Taiwanese population. Further investigation is needed to see if this association is mediated by mere adiposity or SDC3 polymorphism is also linked with other components of MetS such as lipid metabolism.

## 1. Introduction

Metabolic syndrome (MetS) poses a major health burden on the general population worldwide. International Diabetes Federation estimated that a quarter of the world's adults have MetS [1]. MetS is defined as comprising of hypertension, dyslipidemia (raised triglyceride and low high-density lipoprotein cholesterol levels), hyperglycemia, and central obesity [2]. It has been found to be a strong predictor of cardiovascular disease, diabetes mellitus, and even all-cause mortality [3–5].

While lifestyle habits are known to be closely linked with the development of the MetS, it is increasingly clear that genetic factor plays an important role in an individual's risk of developing the MetS. The genetic involvement in MetS was shown in previous familial genetic studies to the more recent genome-wide association studies [6–11].

Syndecan-3 (SDC3), a heparin sulfate proteoglycan, had been found by previous studies to be linked with energy balance and obesity [12–14], but its association with MetS is not known. This study aims to investigate SDC3 polymorphism and its association with MetS in the Taiwanese population.

## 2. Subjects and Methods

### 2.1. Study Population

We recruited 545 subjects aged 20–65 years old in this population-based study. Using the MetS criteria with cut-off values proposed by Taiwan's Ministry of Health and Welfare, there were a total of 154 subjects with MetS and 391 subjects without MetS. The exclusion criteria include (1) pregnancy, (2) cancer, (3) secondary obesity, (4) hereditary disease (such as Prader-Willi syndrome, Bardet-Biedl syndrome), and (5) body mass index (BMI) < 27 kg/m^2^ following bariatric surgery or use of pharmacologic agents for weight reduction. Written informed consent was obtained from each subject. This study was approved by the institutional review board of Mackay Memorial Hospital (number 12MMHIS106).

Anthropometric measurements (including body height (BH), body weight (BW), waist circumference (WC), percent fat mass (PFM), and blood pressure (BP)) were measured for each subject. BMI was calculated using weight in kilograms divided by the square of height in meters. Obesity was defined as BMI ≥ 27 kg/m^2^ by Taiwan's Ministry of Health and Welfare.

Fasting blood specimens were drawn, and biochemical markers including triglyceride (TG), high-density lipoprotein cholesterol (HDL-C), fasting plasma glucose (FPG), insulin, homeostatic model assessment for insulin resistance (HOMA-IR), and high-sensitivity C-reactive protein (hs-CRP) were analyzed by a biochemical autoanalyzer (Beckman Coulter, CA, USA).

MetS was defined as having at least three of the following: large WC (male ≥ 90 cm or female ≥ 80 cm), high BP (systolic blood pressure (SBP) ≥ 130 mmHg or diastolic blood pressure (DBP) ≥ 85 mmHg), low HDL-C (male < 40 mg/dl or female < 50 mg/dl), high FPG (≥100 mg/dl), and high TG (≥150 mg/dl). The cut-off values used for the definition of large WC were given by Taiwan's Ministry of Health and Welfare.

### 2.2. Genotyping

Buccal swabs were collected from each subject using standard protocols, and DNA was isolated using the Isohelix Buccal DNA isolation kit (Cell Projects, Kent, UK) as per manufacturer's instructions. DNA was then purified and concentrated using the DNA Clean & Concentrator kit (Zymo Research, Irvine, CA, USA). The qualities of isolated genomic DNAs were checked using the agarose gel electrophoresis and the quantities were determined using spectrophotometry. Genotyping of SDC3 single nucleotide polymorphism (SNP) was performed using the Taqman SNP genotyping assay. The primers and probes of SNP were from ABI assay on demand kit (ABI: Applied Biosystems Inc., Foster City, CA, USA). Reactions were carried out according to the manufacturer's protocol. The probe fluorescence signal detection was performed using the ABI StepOnePlus™ Real-Time PCR Systems.

### 2.3. Statistical Analysis

SPSS software version 21.0 was used for all statistical analyses. Chi-square and Student's *t*-tests were used to compare characteristics between subjects. Genotype frequencies were evaluated for Hardy-Weinberg equilibrium using a *χ*^2^ goodness-of-fit test. Analysis of covariance (ANCOVA) was used to compare clinical variable mean values, while adjusting for the covariates of age and gender. Odds ratios and their 95% confidence intervals were evaluated. Association between SNP and MetS was tested via logistic regression analysis at the 5% level of significance.

## 3. Results

Descriptive characteristics of the study participants were shown in Table 1. The MetS group showed a higher frequency for SDC3 rs2282440 TT homozygote than the non-MetS group (33.1% versus 20.7%, *p* = 0.0093) (Table 2).

Cardiometabolic factors were compared among the genotypes of SDC3 rs2282440 polymorphism (Table 3**)**. Subjects with SDC3 rs2282440 TT homozygote were shown to have a higher mean BMI (*p* = 0.0132), mean WC (*p* = 0.039), mean PFM (*p* = 0.0139), and mean TG level (*p* = 0.0407) than subjects with CC or CT genotypes. Although no significant difference was found in the mean homa-IR and hs-CRP values among the three genotypes, a higher percentage of subjects with SDC3 rs2282440 TT homozygote had homa-IR values greater than the 75th percentile (*p* = 0.0437) and hs-CRP values greater than the 75th percentile (*p* = 0.0205) as compared with subjects of the other two genotypes. In addition, SDC3 rs2282440 polymorphism was associated with obesity (*p* = 0.0058).

MetS and its five individual components were analyzed among the genotypes of SDC3 rs2282440 polymorphism (Table 4). Subjects with SDC3 rs2282440 TT homozygote had higher frequency of having MetS than those with CC or CT genotype (TT 38.6% versus CC 26.1% versus CT 24.5%; *p* = 0.0217). As for the individual components, subjects with SDC3 rs2282440 TT homozygote had higher frequency of having large WC, high TG, low HDL-C, and high BP. However, only the WC (*p* = 0.0325) and the BP (*p* = 0.0305) components reached statistical significance.

In Table 5, we calculated odds ratios (ORs) for SDC3 rs2282440 polymorphism and its association with MetS and related risk factors after adjustment for sex, age, smoking, alcohol drinking, and exercise habits. Subjects with SDC3 rs2282440 TT homozygote had a 1.96-fold risk of being obese and 1.8-fold risk of having MetS (with CC homozygote as reference). As for the individual components, SDC3 rs2282440 TT homozygote was associated with large WC and low HDL (OR = 1.75 and OR = 1.84, resp.). In addition, subjects with SDC3 rs2282440 TT homozygote was more likely to have hs-CRP value greater than the 75th percentile (OR = 2.0).

## 4. Discussion

Our present study found TT homozygote of SDC3 rs2282440 polymorphism to be associated with MetS susceptibility. Large-scale genome-wide association studies have helped identify common genetic variation associated with MetS. However, to our knowledge, this is the first study to find positive association of SDC3 polymorphism with MetS.

Syndecans are members of the family of heparan sulfate proteoglycans (HSPGs) and are found on all mammalian cell surfaces. HSPGs are unique in their ability to interact with multiple diverse ligands and are mainly involved in morphogenesis, tissue repair, host defense, and energy metabolism [15, 16]. Syndecan family members (SDC 1-4) are differentially expressed in a tissue-specific manner, with SDC3 found primarily on neural crest derivatives and neurons. Expression of syndecans is induced during development and injury, as well as other physiological stimuli [15]. Studies on SDC3 function had discovered its involvement in chondrocyte proliferation, maturation, and function in the developing skeleton [17] and in regulation of energy balance [18].

SDC3 had been well-documented by previous studies to be associated with control of energy balance and obesity [12–14, 19, 20]. SDC3 is expressed in the hypothalamus, which is known to regulate feeding behavior and body weight. Strong association between the SDC3 SNP rs2282440 and obesity had been shown in a Korean study [14] and also confirmed in the Taiwanese population [21].

Many genetic variants involved in the pathogenesis of the metabolic syndrome had been found to be associated with lipid metabolism, namely, SNPs in the APOA5, APOC3, and CETP genes [6, 7, 11, 22–24]. In a Korean study, APOA5 and APOE had significant association with MetS and its components [22]. APOA5 was found to be significantly associated with an increased serum concentration of TG, a decreased serum concentration of HDL-C, and the prevalence of MetS in the Japanese population [23]. In our study, SDC3 polymorphism was found to be associated with MetS components of large WC (OR = 1.75) and low HDL-C level (OR = 1.84). The association with large WC could be explained by the role of SDC3 in regulating body weight. However, the role of SDC3 in lipid metabolism had yet to be elucidated. SDC3 has been found to facilitate agouti-related peptide (AgRP) in antagonizing melanocortin-4 receptor (MC4R) leading to increased body weight [18, 25–27]. Studies had also shown MC4R polymorphism to be related to changes in lipid metabolism [28–30]. Most recently, a Japanese study showed positive association of a common polymorphism near MC4R gene with lipid metabolism [30]. However, further research would be needed to establish the true relationship between SDC3 and lipid metabolism and whether the association is linked to its relation with MC4R-related function or through other pathways.

Insulin resistance is believed to be a main trait in people with MetS and is associated with obesity and diabetes mellitus. In the Strader et al. study [13], SDC3 null mice on high-fat diet were found to have improved glucose tolerance and lower fasting insulin concentrations compared with those of wild-type mice. However, in our study, SDC3 polymorphism was not associated with the fasting plasma glucose level or the glucose component of MetS. Meanwhile, we did find a significantly greater percentage of subjects with SDC3 rs2282440 TT homozygote to have homa-IR values greater than the 75th percentile. Nevertheless, the association of SDC3 with glucose metabolism is most likely to be mediated by its role in body fat regulation.

MetS is considered a low-grade inflammatory condition, and C-reactive protein (CRP) is the best characterized biomarker of inflammation [31, 32]. Hs-CRP has been shown to have significant association with the risk of cardiovascular disease [33]. A prospective cohort found that the level of CRP added clinically prognostic information to future cardiovascular events among those with MetS [34]. In our study, a higher percentage of subjects with SDC3 rs2282440 TT homozygote had hs-CRP values greater than the 75th percentile, and this association stayed true even after adjusting for various confounding factors. This finding further supported the association between SDC3 polymorphism and MetS.

The main limitation of this study was the disproportionate number of subjects in the MetS and non-MetS group because this was the second analysis of the data from a previous obesity study. However, we were able to show that subjects with SDC3 rs2282440 TT homozygote had higher frequency of having MetS even after adjusting for confounding factors, such as sex, age, smoking, alcohol drinking, and exercise habits.

## 5. Conclusion

SDC3 rs2282440 polymorphism is shown to be positively associated with MetS in the Taiwanese population. To our knowledge, this is the first report to demonstrate association of SDC3 polymorphism and MetS. Further investigation is needed to confirm this relationship and see if this association is mediated by mere adiposity or SDC3 polymorphism can also be linked with changes in lipid metabolism.

## Figures and Tables

**Table 1:** Descriptive characteristics of the study participants according to presence of metabolic syndrome.

	Non-MetS group *n* = 391	MetS group *n* = 154	*p*
Age (yrs)	36.8	40.8	<0.0001
Women, *n* (%)	274 (70.1)	69 (44.8)	<0.0001
Smoking, *n* (%)	21 (5.4)	34 (22.1)	<0.0001
Exercise habits, *n* (%)	205 (52.4)	73 (47.4)	0.2905
Alcohol drinking, *n* (%)	54 (13.1)	34 (22.1)	0.0182
Hx of HTN, *n* (%)	34 (8.7)	56 (36.4)	<0.0001
Hx of DM, *n* (%)	5 (1.3)	38 (24.7)	<0.0001
BMI (kg/m^2^)	25.0	30.8	<0.0001
WC (cm)	79.2	97.3	<0.0001
PFM (%)	31.0	36.8	<0.0001
SBP (mmHg)	119.4	136.9	<0.0001
DBP (mmHg)	74.9	86.2	<0.0001
TG (mg/dl)	86.8	185.4	<0.0001
HDL-C (mg/dl)	60.0	43.8	<0.0001
FPG (mg/dl)	88.3	114.0	<0.0001
Insulin (*μ*U/ml)	8.3	18.8	<0.0001
Homa-IR	1.8	5.6	<0.0001
HOMA-IR group			
≥ 75%tile, *n* (%)	68 (17.4)	104 (67.5)	<0.0001
hs-CRP	0.18	0.30	<0.0001
hs-CRP group			
≥ 75%tile, *n* (%)	79 (20.2)	74 (48.1)	<0.0001

MetS: metabolic syndrome; BMI: body mass index; WC: waist circumference; PFM: percent fat mass; SBP: systolic blood pressure; DBP: diastolic blood pressure; TG: triglyceride; HDL-C: high-density lipoprotein cholesterol; FPG: fasting plasma glucose; HOMA-IR indicates homeostasis model assessment for insulin resistance; hs-CRP: high-sensitivity C-reactive protein.

**Table 2:** Genotype frequencies of SDC3 polymorphism (rs2282440) according to the presence of metabolic syndrome.

	Non-MetS group *n* = 391	MetS group *n* = 154	*p*
SDC3(rs2282440)			
C/C, *n* (%)	88 (22.5)	31 (20.1)	0.0093
C/T, *n* (%)	222 (56.8)	72 (46.8)	
T/T, *n* (%)	81 (20.7)	51 (33.1)	

MetS: metabolic syndrome.

**Table 3:** Anthropometric and cardiometabolic measures according to SDC3 polymorphism (rs2282440) genotypes.

	C/C	C/T	T/T	*p*
	*n* = 119	*n* = 294	*n* = 132	
Age, yrs	39.2	37.2	38	0.2834
Sex (women), %	65.6	64.3	57.6	0.3324
BMI, kg/m^2^	26.0	26.4	27.9	**0.0132**
WC, cm	82.8	83.5	87.4	**0.039**
PFM, %	31.9	32.3	34.0	**0.0139**
SBP, mmHg	122.5	123.9	127.2	0.14
DBP, mmHg	77.1	77.8	79.7	0.3282
TG, mg/dl	105.7	110.9	131.1	**0.0407**
HDL-C, mg/dl	57.4	56.1	52.2	0.0539
FPG, mg/dl	96.9	93.8	98.4	0.1857
HOMA-IR	2.6	2.9	3.3	0.2287
HOMA-IR group				
≥ 75%tile, *n* (%)	39 (32.8)	80 (27.2)	53 (40.2)	**0.0437**
hs-CRP	0.21	0.21	0.21	0.1374
hs-CRP group				
≥ 75%tile, *n* (%)	27 (22.7)	77 (26.2)	49 (37.1)	**0.0205**
Obesity, *n* (%)	68 (57.1)	162 (55.1)	96 (72.7)	**0.0058**

*p* values were calculated by using the Proc CATMOD test for categorical variables after adjustment for sex and general linear models for continuous variables after adjustment for sex and age. BMI: body mass index; WC: waist circumference; PFM: percent fat mass; SBP: systolic blood pressure; DBP: diastolic blood pressure; TG: triglyceride; HDL-C: high-density lipoprotein cholesterol; FPG: fasting plasma glucose; HOMA-IR indicates homeostasis model assessment for insulin resistance; hs-CRP: high-sensitivity C-reactive protein.

**Table 4:** Metabolic syndrome and its individual components according to SDC3 polymorphism (rs2282440) genotypes.

	C/C	C/T	T/T	*p*
	*n* = 119	*n* = 294	*n* = 132	
MetS, *n* (%)	31 (26.1)	72 (24.5)	51 (38.6)	**0.0217**
Large WC, *n* (%)	52 (43.7)	130 (44.2)	77 (58.3)	**0.0325**
High TG, *n* (%)	33 (27.7)	75 (25.5)	42 (31.8)	0.5251
Low HDL-C, *n* (%)	25 (21.0)	74 (25.2)	44 (33.3)	0.0946
High BP, *n* (%)	49 (41.2)	104 (35.4)	66 (50.0)	**0.0305**
High FPG, *n* (%)	27 (22.7)	55 (18.7)	30 (22.7)	0.5579

*p* values were calculated by using the Proc CATMOD test for categorical variables after adjustment for sex. MetS: metabolic syndrome; WC: waist circumference; TG: triglyceride; HDL-C: high-density lipoprotein cholesterol; BP: blood pressure; FPG: fasting plasma glucose.

**Table 5:** Relationship between SDC3 polymorphism (rs2282440) and metabolic syndrome and related risk factors.

	C/C	C/T	T/T
	OR (95% CI)	OR (95% CI)	OR (95% CI)
MetS	1.00 (reference)	0.92 (0.54–1.55)	**1.80 (1.01–3.22)**
Large WC	1.00 (reference)	1.00 (0.64–1.57)	**1.75 (1.04–2.96)**
High TG	1.00 (reference)	0.93 (0.56–1.55)	1.20 (0.67–2.13)
Low HDL-C	1.00 (reference)	1.26 (0.74–2.13)	**1.84 (1.02-3.32)**
High BP	1.00 (reference)	0.78 (0.49–1.24)	1.39 (0.82–2.36)
High FPG	1.00 (reference)	0.87 (0.50–1.52)	1.05 (0.56–1.98)
Obesity	1.00 (reference)	0.88 (0.55–1.4)	**1.96 (1.11–3.45)**
HOMA-IR group			
≥ 75%tile	1.00 (reference)	0.74 (0.46–1.19)	1.31 (0.77–2.25)
hs-CRP group			
≥ 75%tile	1.00 (reference)	1.17 (0.70–1.94)	**2.00 (1.15–3.51)**

Odds ratios were calculated by using multiple logistic regression models for categorical SDC3 genotypes after adjustment for sex, age, smoking, alcohol drinking, and exercise habits. MetS: metabolic syndrome; WC: waist circumference; TG: triglyceride; HDL-C: high-density lipoprotein cholesterol; BP: blood pressure; FPG: fasting plasma glucose; HOMA-IR indicates homeostasis model assessment for insulin resistance; hs-CRP: high-sensitivity C-reactive protein.

## References

[1] Alberti K. G., Eckel R. H., Grundy S. M. (2009). Hational Heart, Lung, and Blood Institute, American Heart Association, World Heart Federation, International Atherosclerosis Society, International Association for the Study of Obesity, Harmonizing the metabolic syndrome: a joint interim statement of the International Diabetes Federation Task Force on Epidemiology and Prevention; National Heart, Lung, and Blood Institute; American Heart Association; World Heart Federation; International Atherosclerosis Society; and International Association for the Study of Obesity. *Circulation*.

[2] International Diabetes Federation The IDF consensus worldwide definition of the metabolic syndrome. http://www.idf.org/metabolic-syndrome.

[3] Kaur J. (2014). A comprehensive review on metabolic syndrome. *Cardiology Research and Practice*.

[4] Holvoet P. (2008). Relations between metabolic syndrome, oxidative stress and inflammation and cardiovascular disease. *Verhandelingen - Koninklijke Academie voor Geneeskunde van België*.

[5] Samson S. L., Garber A. J. (2014). Metabolic syndrome. *Endocrinology and Metabolism Clinics of North America*.

[6] Povel C. M., Boer J. M. A., Reiling E., Feskens E. J. (2011). Genetic variants and the metabolic syndrome: a systematic review. *Obesity Reviews*.

[7] Kristiansson K., Perola M., Tikkanen E. (2012). Genome-wide screen for metabolic syndrome susceptibility loci reveals strong lipid gene contribution but no evidence for common genetic basis for clustering of metabolic syndrome traits. *Circulation Cardiovascular Genetics*.

[8] Fall T., Ingelsson E. (2014). Genome-wide association studies of obesity and metabolic syndrome. *Molecular and Cellular Endocrinology*.

[9] Kraja A. T., Vaidya D., Pankow J. S. (2011). A bivariate genome-wide approach to metabolic syndrome: STAMPEED consortium. *Diabetes*.

[10] Tekola-Ayele F., Doumatey A. P., Shriner D. (2015). Genome-wide association study identifies African-ancestry specific variants for metabolic syndrome. *Molecular Genetics and Metabolism*.

[11] Zabaneh D., Balding D. J. (2010). A genome-wide association study of the metabolic syndrome in Indian Asian men. *PLoS One*.

[12] Reizes O., Lincecum J., Wang Z. (2001). Transgenic expression of syndecan-1 uncovers a physiological control of feeding behavior by syndecan-3. *Cell*.

[13] Strader A. D., Reizes O., Woods S. C., Benoit S. C., Seeley R. J. (2004). Mice lacking the syndecan-3 gene are resistant to diet-induced obesity. *The Journal of Clinical Investigation*.

[14] Ha E., Kim M. J., Choi B. K. (2006). Positive association of obesity with single nucleotide polymorphisms of syndecan 3 in the Korean population. *The Journal of Clinical Endocrinology & Metabolism*.

[15] Bernfield M., Götte M., Park P. W. (1999). Functions of cell surface heparan sulfate proteoglycans. *Annual Review of Biochemistry*.

[16] Park P. W., Reizes O., Bernfield M. (2000). Cell surface heparan sulfate proteoglycans: selective regulators of ligand-receptor encounters. *The Journal of Biological Chemistry*.

[17] Pacifici M., Shimo T., Gentili C. (2005). Syndecan-3: a cell-surface heparan sulfate proteoglycan important for chondrocyte proliferation and function during limb skeletogenesis. *Journal of Bone and Mineral Metabolism*.

[18] Reizes O., Clegg D. J., Strader A. D. (2006). A role of syndecan-3 in the melanocortin regulation of energy balance. *Peptides*.

[19] Reizes O., Benoit S. C., Clegg D. J. (2008). The role of syndecans in the regulation of body weight and synaptic plasticity. *The International Journal of Biochemistry & Cell Biology*.

[20] Bellin R., Capilla I., Lincecum J., Park P. W., Reizes O., Bernfield M. R. (2002). Unlocking the secrets of syndecans: transgenic organisms as a potential key. *Glycoconjugate Journal*.

[21] Huang W. H., Hwang L. C., Chan H. L., Lin H. Y., Lin Y. H. (2016). Study of seven single-nucleotide polymorphisms identified in East Asians for association with obesity in a Taiwansese population. *BMJ Open*.

[22] Son K. Y., Son H. Y., Chae J. (2015). Genetic association of *APOA5* and *APOE* with metabolic syndrome and their interaction with health-related behavior in Korean men. *Lipids in Health and Disease*.

[23] Yamada Y., Ichihara S., Kato K. (2008). Genetic risk for metabolic syndrome: examination of candidate gene polymorphisms related to lipid metabolism in Japanese people. *Journal of Medical Genetics*.

[24] Carty C. L., Bhattacharjee S., Haessler J. (2014). Analysis of metabolic syndrome components in >15000 African Americans identifies pleiotropic variants: results from the population architecture using genomics and epidemiology study. *Circulation Cardiovascular Genetics*.

[25] Reizes O., Benoit S. C., Strader A. D., Clegg D. J., Akunuru S., Seeley R. J. (2003). Syndecan-3 modulates food intake by interacting with the melanocortin/AgRP pathyway. *Annals of the New York Academy of Sciences*.

[26] Karlsson-Lindahl L., Schmidt L., Haage D. (2012). Heparanase affects food intake and regulates energy balance in mice. *PLoS One*.

[27] Zheng Q., Zhu J., Shanabrough M. (2010). Enhanced anorexigenic signaling in lean obesity resistance syndecan-3 null mice. *Neuroscience*.

[28] Bronner G., Sattler A. M., Hinney A. (2006). The 103I variant of the melanocortin 4 receptor is associated with low serum triglyceride levels. *The Journal of Clinical Endocrinology & Metabolism*.

[29] Nogueiras R., Wiedmer P., Perez-Tilve D. (2007). The central melanocortin system directly controls peripheral lipid metabolism. *The Journal of Clinical Investigation*.

[30] Katsuura-Kamano S., Uemura H., Arisawa K. (2014). A polymorphism near *MC4R* gene (rs17782313) is associated with serum triglyceride levels in the general Japanese population: the J-MICC study. *Endocrine*.

[31] Elks C. M., Francis J. (2010). Central adiposity, systemic inflammation, and the metabolic syndrome. *Current Hypertension Reports*.

[32] Devaraj S., Valleggi S., Siegel D., Jialal I. (2010). Role of C-reactive protein in contributing to increased cardiovascular risk in metabolic syndrome. *Current Atherosclerosis Reports*.

[33] Koenig W. (2013). High-sensitivity C-reactive protein and atherosclerotic disease: from improved risk prediction to risk-guided therapy. *International Journal of Cardiology*.

[34] Ridker P. M., Buring J. E., Cook N. R., Rifai N. (2003). C-reactive protein, the metabolic syndrome, and risk of incident cardiovascular events: an 8-year follow-up of 14719 initially healthy Amercian women. *Circulation*.

